# Genetic analysis of potential markers and therapeutic targets for immunity in periodontitis

**DOI:** 10.3389/fdmed.2024.1480346

**Published:** 2024-11-22

**Authors:** Hui Li, Wanqing Du, Xin Ye, Xi Luo, Xuejing Duan

**Affiliations:** ^1^ School of Stomatology, Shandong First Medical University, Jinan, China; ^2^ Department of Stomatology, Shandong Provincial Hospital Affiliated to Shandong First Medical University, Jinan, China

**Keywords:** periodontitis, immune-related genes, diagnostic model, marker genes, immunity

## Abstract

**Objective:**

Periodontitis is a chronic inflammatory periodontal disease resulting in destroyed periodontal tissue. Many studies have found that the host's inflammatory immune responses are involved in the risk of periodontal tissue damage. In this study, we aim to identify potential biomarkers and therapeutic targets related to immunity in periodontitis.

**Methods:**

GSE16134 and GSE10334 were downloaded from the Gene Expression Omnibus (GEO) database, and the immune-related genes were obtained from the Immunology Database and Analysis Portal (ImmPort). After the differentially expressed immune-related genes (DE-IRGs) were identified, enrichment analysis was performed. Two machine learning methods, the least absolute shrinkage and selector operation (LASSO) logistic regression and the support vector machine-recursive feature elimination (SVM-RFE), were used to screen out potential markers for the diagnosis of periodontitis. The CIBERSORT algorithm and LM22 matrix were used to analyze the percentage of infiltrating immune cells in periodontitis. Finally, the potential drug targets for the selected immune-related marker genes were predicted using relevant databases.

**Results:**

A total of 7 genes (CD19, CXCR4, FABP4, FOS, IGHD, IL2RG, and PPBP) were upregulated in periodontitis samples. The area under the receiver operating characteristic curve (AUC) value of only one gene for distinguishing periodontitis from healthy samples ranged from 0.724 to 0.894. The prediction ability of the combined risk score of these 7 DE-IRGs was improved (AUC = 0.955). Naïve B cells, neutrophils, plasma cells, and activated memory CD4 T cells were significantly enriched in periodontitis samples, and 25 drugs targeting 4 DE-IRGs were predicted.

**Conclusion:**

We developed a diagnostic model based on seven IRGs for periodontitis. The possible drugs targeting IRGs may provide new ideas for periodontitis treatment.

## Introduction

Periodontitis is currently the sixth most prevalent disease among adults worldwide (1). Periodontitis is a bacterially induced inflammatory disease that slowly destroys the connective tissue and bone-supporting teeth in the oral cavity. The initiating factor of periodontal disease is dental plaque microorganisms, which is a major contributing factor in periodontal disease (2). Furthermore, the development of periodontitis is also affected by local and systemic risk factors, among which are dental calculus, occlusal trauma, smoking, diabetes mellitus, etc. Recent studies have uncovered novel mechanisms underlying the breakdown of periodontal hostmicrobe homeostasis, which can precipitate dysbiosis and periodontitis in susceptible hosts. In periodontal diseases, polymicrobial communities induce a dysregulated and destructive host response through an overall mechanism referred to as polymicrobial synergy and dysbiosis (3, 4). The main treatment methods for periodontitis are basic and surgical therapies, which mainly aim to remove plaque, calculus, the inner wall, and root surface of infected pockets, in order to completely eliminate periodontal inflammation (5). The former encompasses scaling, subgingival scaling and root planning, plaque control, adjunctive therapy with antibacterial drugs, etc. The latter comprises periodontal resection and periodontal regeneration. Additionally, there are some new technologies, such as the application of lasers and bioactive factors.

Periodontal pathogens can escape from the host immune reaction through various mechanisms and thus suppress the innate immune response of oral epithelial cells, which results in persistent periodontal infection (6). Chronic inflammation caused by the subgingival microbial community has been identified as the main reason for periodontitis and alveolar bone resorption (7). The keystone-pathogen hypothesis holds that certain low-abundance microbial pathogens can orchestrate inflammatory disease by remodelling a normally benign microbiota into a dysbiotic one (8). Porphyromonas gingivalis is a key pathogen in periodontitis that reshapes commensal microbiota to dysbiotic partners and destroys the stable balance with the host tissue, resulting in destructive inflammation (8, 9). This partially explains the complex etiology of periodontitis. In addition, the dysbiotic oral microbial communities could mediate inflammatory pathology (10). Periodontal bacteria can influence the interaction with the host immune reaction to increase their adaptability (10). Chronical exposure to dysbiotic microbial communities that evade the host immune response and promote inflammation can adversely affect systemic health.

At present, the molecular mechanisms of periodontitis are not fully understood, but it is generally believed that they involve complex interactions among multiple factors. These include the release of inflammatory mediators, imbalanced regulation of cytokines, abnormal host immune responses, the effects of microorganisms and their toxins on cell signaling pathways, and individual susceptibility differences due to genetic factors. The specific molecular mechanisms remain incompletely clear. Recent advancements in bioinformatics techniques have opened new avenues for understanding the underlying genetic and molecular frameworks associated with periodontitis. By employing high-throughput sequencing technologies and microarray analyses, researchers are able to identify differentially expressed genes that may play critical roles in the pathogenesis of this condition. These genes could be involved in various biological processes such as cell signaling pathways, immune responses, and tissue remodeling. The use of bioinformatics techniques to investigate the differentially expressed genes related to periodontitis and their possible functions and signaling pathways may offer a bioinformatics foundation for the diagnosis, treatment and prognosis of periodontitis. Furthermore, integrating bioinformatics approaches with clinical data could enhance our ability to develop biomarkers for early diagnosis and prognosis of periodontitis. Identifying periodontitis at an early stage of chronic inflammatory responses greatly improves the prognosis because the alveolar bone resorption is irreversible at a later stage (11). A number of studies have employed bioinformatics techniques in the diagnosis and prognosis of periodontal diseases. For instance, some research has discovered diagnostic markers based on miRNAs and cell pyroptosis-related genes, whose sensitivity and specificity are convincingly applicable for the diagnosis of periodontal diseases (12, 13), suggesting the potential application of bioinformatics in diagnosis and prognosis of periodontitis.

As previously stated, inflammatory immune responses are correlated with the risk of periodontal tissue damage. Bacteria trigger an immune response within the host's body, leading to inflammatory cell infiltration. The host's immune cells play dual roles in chronic periodontitis, including controlling periodontal infections and destroying periodontal tissues. Numerous genes or cytokines play different roles in the process. Hence, through analyzing the relationship between periodontitis and immune genes, we can acquire a deeper understanding of this disease and apply it to subsequent diagnosis and treatment. Here we systematically explored the expression of immune-related genes (IRGs) and constructed an IRGs-based diagnostic signature to analyze the relationship between the expression of IRGs and the level of immune cell infiltration. Furthermore, identifying possible drugs targeting these IRGs could provide valuable insights into novel treatment options for periodontitis management. These therapeutics might include small molecules or biologics designed specifically to modulate key signaling pathways associated with inflammation and immunity within periodontal tissues. Ultimately, integrating findings from our research into clinical practice has the potential to enhance diagnostic accuracy and improve patient outcomes through personalized medicine approaches tailored for individuals suffering from chronic periodontitis.

## Materials and methods

### Data acquisition and pre-processing

Microarray data GSE16134 (14) and GSE10334 (15) were download from the Gene Expression Omnibus (GEO) database (http://www.ncbi.nlm.nih.gov/geo). GSE16134 dataset contained 241 periodontitis samples of gingival tissues and 69 healthy samples, while GSE10334 dataset contained 183 periodontitis samples and 64 healthy samples. Periodontitis samples in two dataset are obtained from patients with chronic or aggressive periodontitis. Both datasets were retrieved from the GPL570 (HG-U133_Plus_2) Affymetrix Human Genome U133 Plus 2.0 Array. The GSE16134 dataset was used as the discovery dataset, while the GSE10334 dataset as the external validation dataset. To pre-process the gene expression data, probes were converted to gene symbols based on the annotation file provided by the platform manufacturer. In particular, probes without a corresponding gene symbol were removed, and average values were calculated when a gene corresponded to multiple probes. A total of 2,483 IRGs were obtained from the Immunology Database and Analysis Portal (ImmPort, https://www.immport.org/) (16). After removing duplicated genes, 1,793 IRGs were finally included in this paper.

### Differential gene analysis and functional enrichment

The differentially expressed genes (DEG) between periodontitis samples and healthy samples were identified using *limma* package version 3.34.7 in R3.6 (17). The inclusion criteria of DEGs included an absolute log2FC > 1 and the BH-adjusted *p* value < 0.05. The Gene Ontology (GO) and Kyoto Encyclopedia of Genes and Genomes (KEGG) enrichment were performed with ClusterProfiler packages 4 (18).

### Identification of IRG diagnostic markers for periodontitis

In the training step, the least absolute shrinkage and selection operator (LASSO) in the R3.6.1 package Version 1.2 penalty was applied to the discovery dataset to build an optimal IRG signature for data dimension reduction (19). Ten-fold cross-validation was performed to find the optimal penalty parameter that gave the maximum AUC value. Meanwhile, the e1071 R3.6.1 package was used to build a support vector machine-recursive feature elimination (SVM-RFE) model and the mean error rate was compared with 10-fold cross-validation (20). Additionally, overlapping genes obtained from both algorithms were considered the best IRG markers for periodontitis. Moreover, using the R package glm, the best gene markers were used to construct the IRG diagnostic signature based on the following formula: Risk Score = $\sum_{i=1}^{n}Coef_{i}\times E_{i}$, where the *Coef_i_* is the coefficient and the *E_i_* is the normalized expression of selected IRGs by log2 and Z-score transformation. Finally, receiver operating characteristic (ROC) curves were plotted, and the AUC was used to evaluate whether the selected gene markers and IRG signature had diagnostic values.

In the validation step, the same formula generated on the discovery dataset was used to calculate the risk score in the external validation dataset and the AUC value was used to evaluate the IRG signature in periodontitis diagnosis.

### Immune cell infiltration

To assess the immune activity, we applied the R3.6.1 package “ESTIMATE” (21) to quantify the infiltrating immune cells in the gingival tissues according to their gene expression values and obtained immune scores for each sample in the GSE16134 dataset.

The CIBERSORT algorithm and LM22 matrix were used to analyze the percentage of infiltrating immune cells in the microenvironment for each sample in the GSE16134 dataset (22). CIBERSORT is a deconvolution algorithm using transcriptomic data of 547 characteristic genes to predict the infiltration of 22 immune cell types in each sample (22).

### Marker gene and drug interactions

To identify potential drugs for periodontitis, relevant databases were used to predict the drug targets of the selected marker genes (23). Over 100,000 drug-gene interactions were collected from DrugBank (24), PharmGKB (25), Chembl (https://www.ebi.ac.uk/chembl/), Drug Target Commons (26), TTD (http://bidd.nus.edu.sg/group/cjttd/), and Drug Gene Interaction Database (DGIdb) (23) and the interactions were visualized with Cytoscape (27).

**Figure 1:**
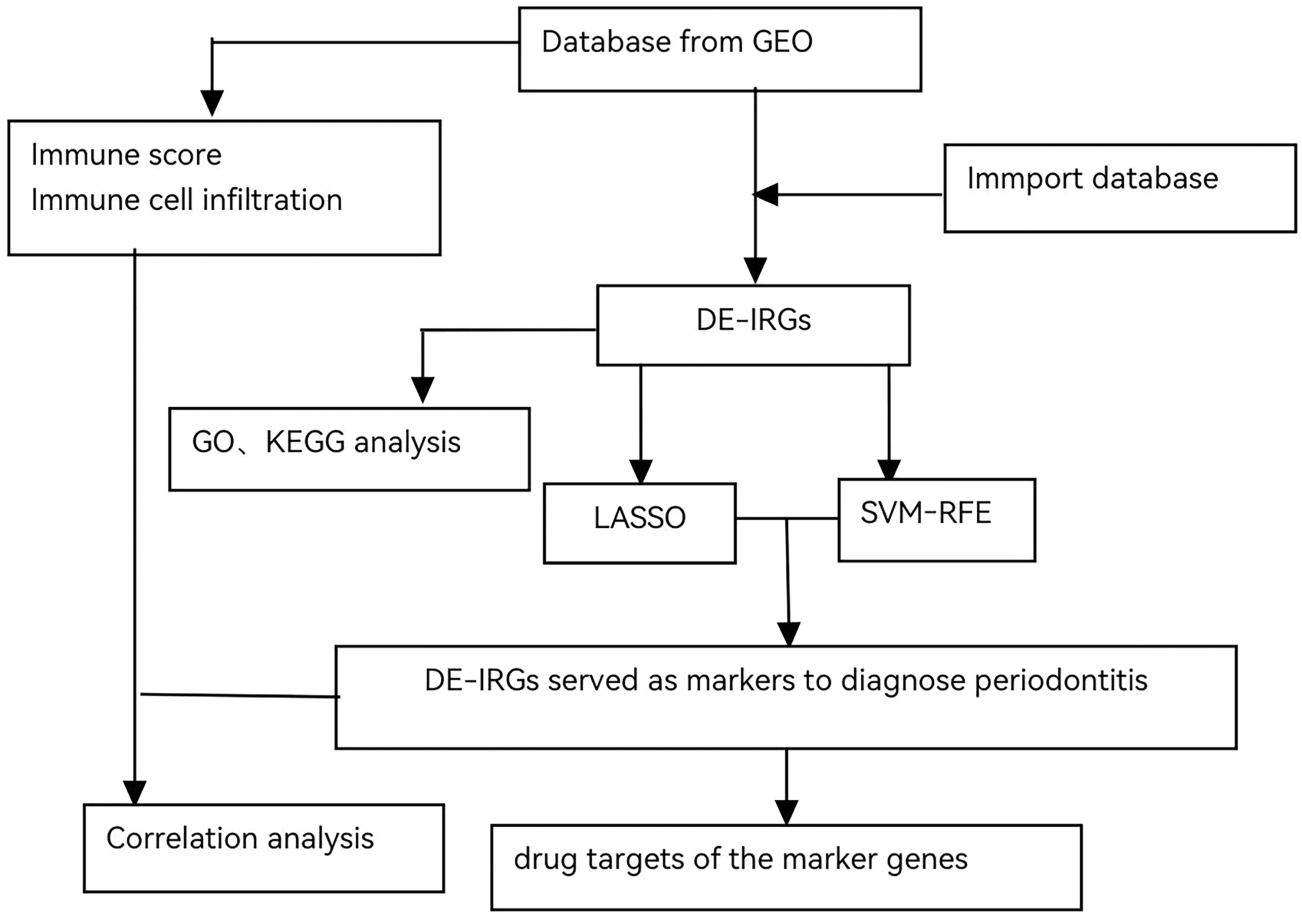
The flowchart of the experimental process.

A comprehensive flowchart was developed to illustrate the entire experimental process (Figure 1).

## Results

### DE-IRGs identified from the GSDE16134 dataset

**Figure 2:**
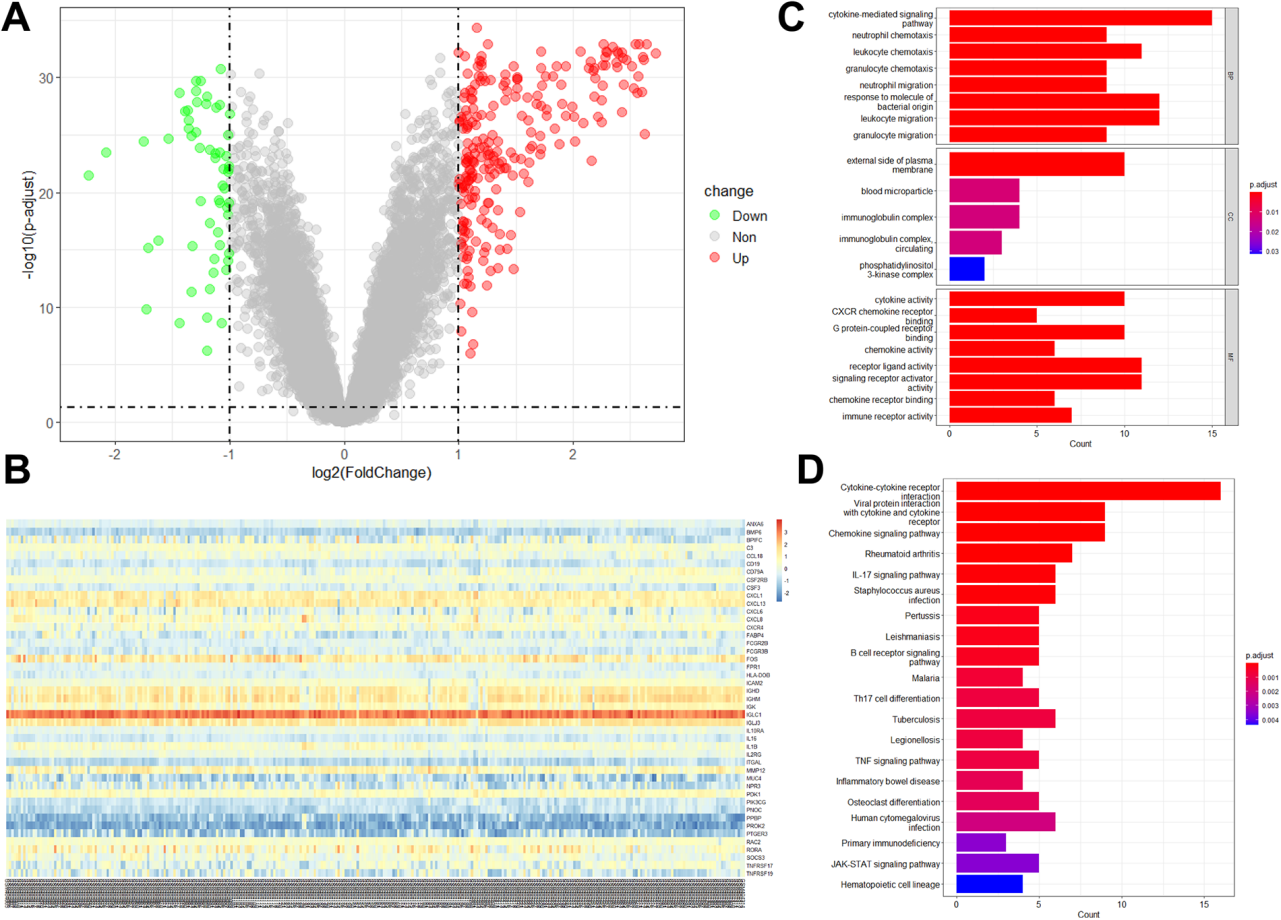
DE-IRGs expression levels in periodontitis samples and functional analyses of DE-IRGs. **(A)** Volcano plot showed the DEGs between periodontitis and healthy samples in the GSE16134 dataset. **(B)** The expression of 45 DE-IRGs in all samples in the GSE16134 dataset. The enrichment analysis showed the enriched GO terms **(C)** and KEGG pathways **(D)**.

There were 234 up-regulated genes and 58 down-regulated genes in periodontitis samples compared with healthy samples (Figure 2A). Among them, 45 differentially expressed immune-related genes (DE-IRGs) were identified, including 40 with up-regulation and 5 with down-regulation (Figure 2B). To elucidate the biological functions of the 45 DE-IRGs, GO and KEGG enrichment analyses were performed. GO-molecular function (MF) revealed the significant enrichment of DE-IRGs in “cytokine activity”, “CXCR chemokine receptor binding”, “G protein-coupled receptor binding”, “chemokine activity”, “receptor ligand activity”, and “immune receptor activity” (Figure 2C). With regard to cellar component (CC), DE-IRGs were significantly associated with “external side of plasma membrane”, “blood microparticle”, and “immunoglobulin complex” (Figure 2C). As for GO-biological process (BP) annotation, DE-IRGs were associated with “cytokine-mediated signaling pathway”, “neutrophil chemotaxis”, “leukocyte chemotaxis”, and “granulocyte chemotaxis” (Figure 2C). The KEGG enrichment analysis suggested the close associations of DE-IRGs with “cytokine-cytokine receptor interaction”, “chemokine signaling pathway”, and “IL-17 signaling pathway” (Figure 2D).

### Seven DE-IRGs served as markers to diagnose periodontitis

**Figure 3:**
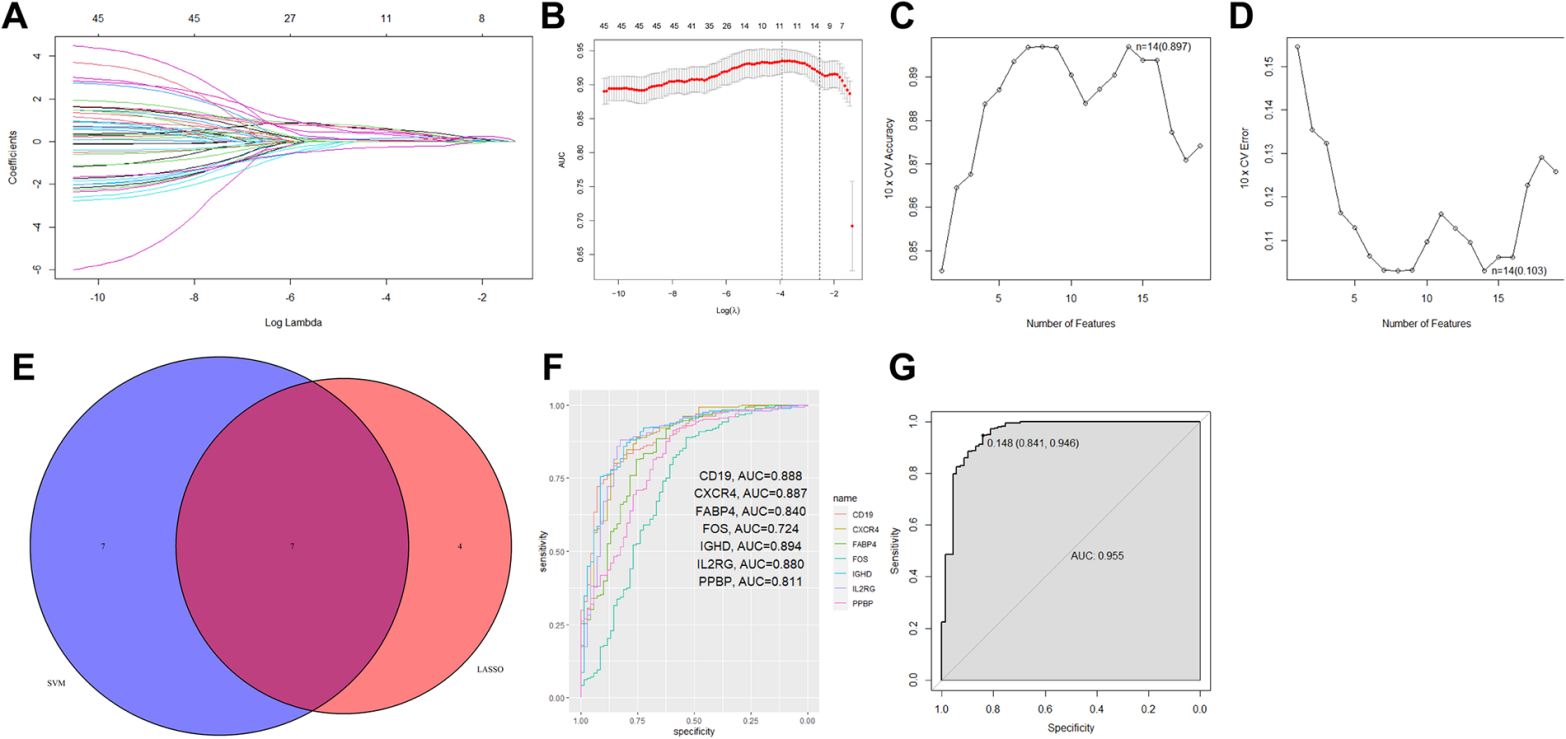
Seven DE-IRGs were identified as diagnostic markers for periodontitis. **(A,B)** Using LOSSO logistic regression method, ten-fold cross-validation was conducted to tune the optimal value of the penalty parameter, and 11 DE-IRGs were identified. **(C,D)** The SVM-RFE algorithm selected the best combine of 14 DE-IRGs in predicting periodontitis. **(E)** The Venn plot showed the overlapping genes identified by the LOSSO logistic regression method and the SVM-RFE algorithm. **(F)** The ROC curve showed the ability to predict periodontitis using single gene. **(G)** The combination of 7 DE-IRGs in a logistic regression model to identify the AUC of periodontitis samples in the GSE16134 dataset.

The use of DE-IRGs in the diagnosis of periodontitis was further investigated. Two different machine learning algorithms LASSO and SVM-RFE were then used to select DE-IRGs in the GSE16134 dataset. LASSO algorithm was performed on the 45 DE-IRGs, and a total of 11 optimized diagnostic DE-IRGs were obtained (Figures 3A,B). Then SVM-RFE algorithm identified 14 DE-IRGs as the optimal combination of feature genes (Figures 3C,D). The 7 overlapping DE-IRGs (CD19, CXCR4, FABP4, FOS, IGHD, IL2RG, and PPBP) were finally identified to serve as diagnostic markers (Figure 3E).

Based on the filtered 7 DE-IRGs, the R package glm was further used to construct the IRG diagnostic signature. The corresponding risk score was computed according to the formula: Risk score = 1.425 × CD19 + 0.447 × CXCR4 + 3.270 × FABP4 + 0.831 × FOS + 0.431 × IGHD + 0.832 × IL2RG + 1.925 × PPBP + 4.046. According to the ROC curve analysis, the AUC value of only one gene for distinguishing periodontitis from healthy samples ranged from 0.724 to 0.894 (Figure 3F). In the combination of all 7 DE-IRGs, the prediction ability of the model was improved (AUC = 0.955) (Figure 3G). Based on the calculated risk score, the patients were assigned into high-risk and low-risk groups according to the cutoff determined by the ROC curve (Supplementary Figure S1). The expression levels of 7 DE-IRGs were displayed in heatmap plot (Supplementary Figure S1).

**Figure 4:**
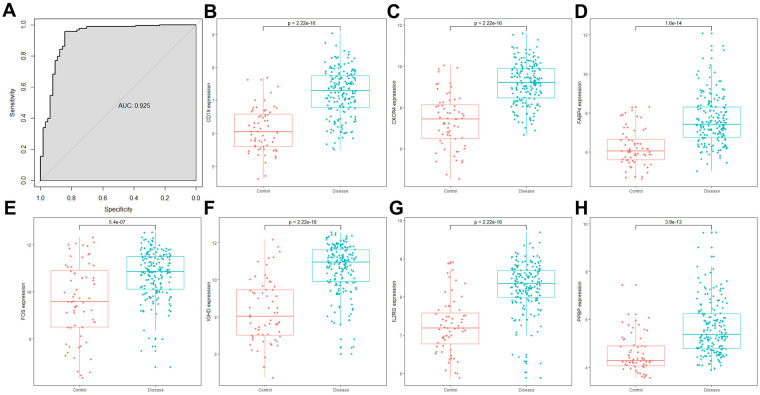
The combined risk score and the expression of 7 DE-IRGs in the GSE10334 dataset. **(A)** The ROC curve showed the ability to predict periodontitis in the GSE10334 dataset. **(A–H)** The expression of 7 DE-IRGs in the GSE10334 dataset.

We further validated the model in the GSE10334 dataset, with the risk score calculated according to the same formula. The ROC curve showed that the combined model had a good performance in distinguishing periodontitis from healthy samples in the external validation dataset (AUC = 0.925, Figure 4A). The expression levels of all diagnostic marker genes were validated in the GSE10334 dataset. All genes showed similar expression pattern to that in the GSE16134 dataset, with significantly higher expression in periodontitis samples (Figures 4B,H).

### Immune microenvironment in periodontitis

**Figure 5:**
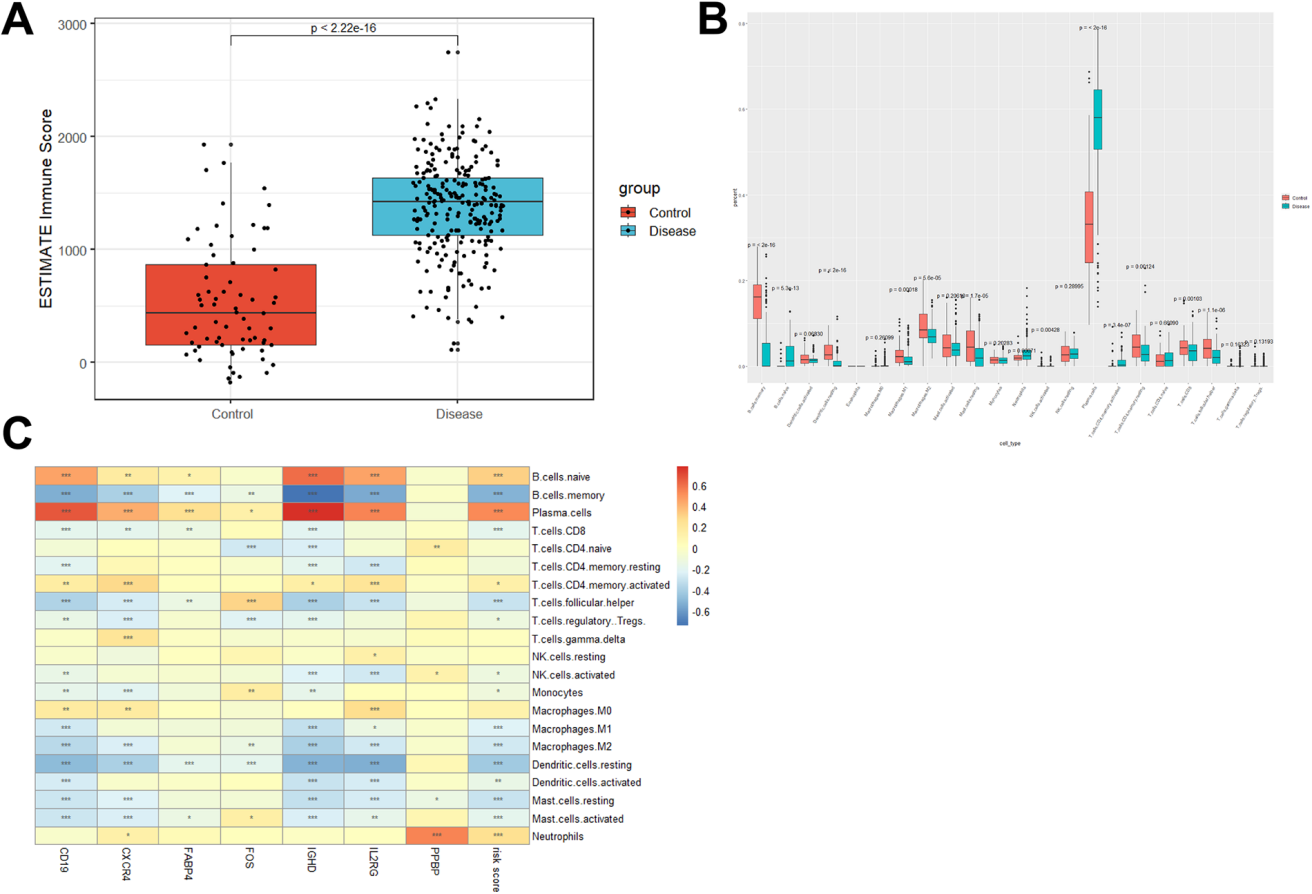
Distinct immune cell infiltration in periodontitis samples and healthy controls. **(A)** Immune score was increased in periodontitis samples compared with healthy controls. **(B)** CIBERSORT was conducted to show the level of 22 immune cell infiltration in periodontitis samples and healthy controls in the GSE16134 dataset. **(C)** The correlation between the expression of 7 DE-IRGs and the percentage of immune cell infiltration.

Evidence suggests that periodontitis is closely associated with the immune microenvironment. Based on the R package Estimate, immune scores for each sample in the GSE16134 dataset were calculated. The periodontitis samples showed higher immune scores than healthy samples (*p* value < 2.26 × 10^−^16, Figure 5A). The infiltration level of 22 immune cell types was further determined with the CIBERSORT algorithm (Figure 5B). After Bonferroni correction (*p* value < 2.3 × hnyt10^−3^, 0.05/22), it was found that naïve B cells, neutrophils, plasma cells, and activated memory CD4 T cells were significantly enriched in periodontitis samples (*p* value < 2.26 × 10^−16^, Figure 5B). While the percentages of memory B cells, activated dendritic cells, M1 macrophages, M2 macrophages, resting mast cells, CD8 T cells, and follicular helper T cells in periodontitis samples were lower than those in healthy samples (Figure 5B). Pearson correlation analyses suggested that naïve B cells had strong positive correlations with the expression of CD19 (*r* = 0.44, *p* value = 2.54 × 10^−16^), CXCR4 (*r* = 0.17, *p* value = 2.64 × 10^−3^), FABP4 (*r* = 0.12, *p* value = 0.036), IGHD (*r* = 0.60, *p* value = 1.78 × 10^−31^), IL2RG (*r* = 0.44, *p* value = 3.42 × 10^−16^), and risk score (*r* = 0.32, *p* value = 1.40 × 10^−8^). The expression of CXCR4 (*r* = 0.13, *p* value = 0.027) and PPBP (*r* = 0.55, *p* value = 1.43 × 10^−25^) and the risk score (*r* = 0.25, *p* value = 1.07 × 10^−5^) were positively correlated with neutrophils. While plasma cells were positively correlated with CD19 (*r* = 0.65, *p* value = 4.35 × 10^−39^), CXCR4 (*r* = 0.42, *p* value = 1.44 × 10^−14^), FABP4 (*r* = 0.25, *p* value = 9.33 × 10^−6^), FOS (*r* = 0.12, *p* value = 0.031), IGHD (*r* = 0.77, *p* value = 3.75 × 10^−63^), IL2RG (*r* = 0.54, *p* value = 3.13 × 10^−25^), and the risk score (*r* = 0.53, *p* value = 5.84 × 10^−24^) (Figure 5C).

### Drugs that may target marker genes were predicted

**Figure 6:**
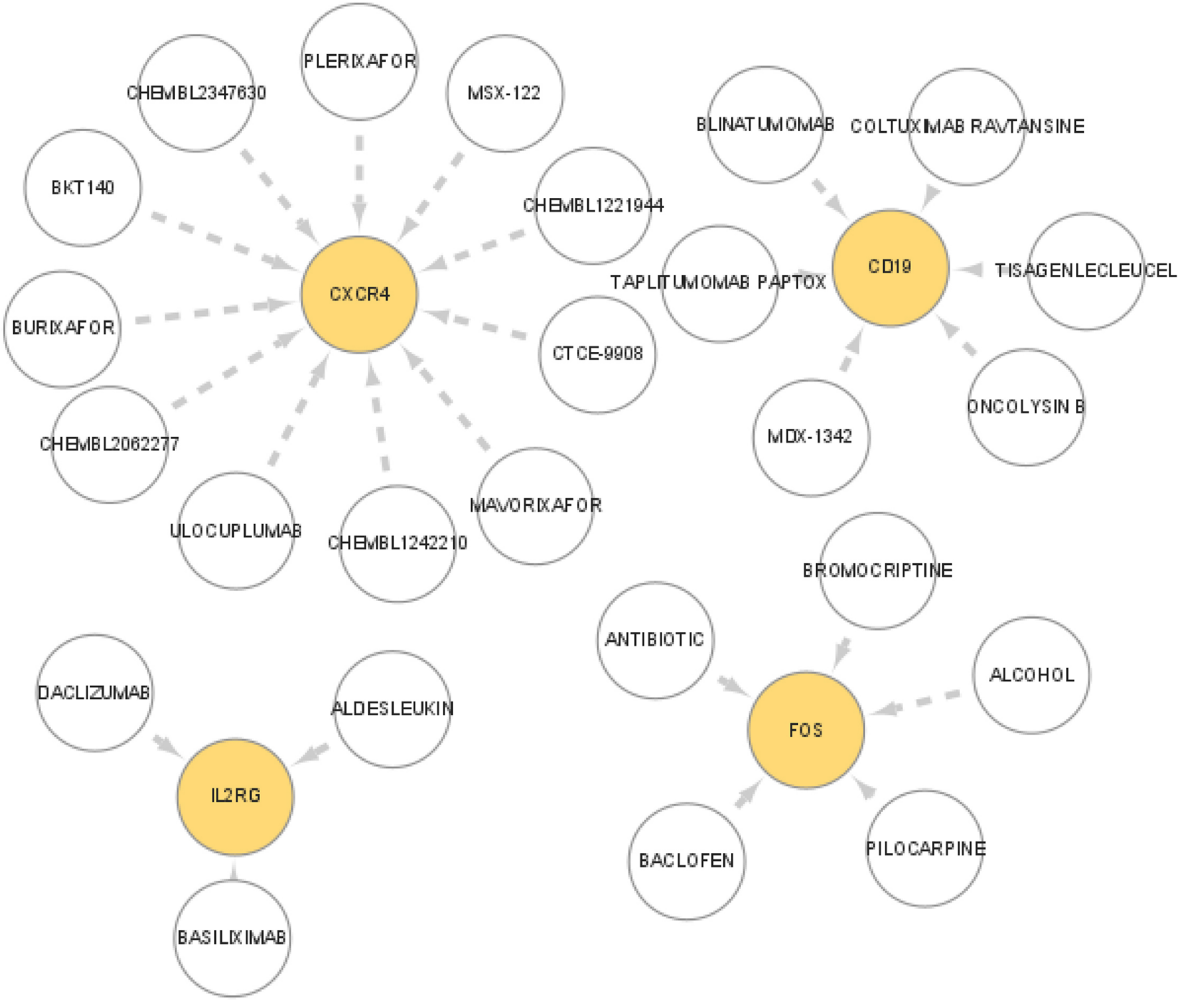
Prediction of the interaction between the marker genes and drugs. The drugs that may target marker genes were predicted though the DGIdb database. The interaction between drugs and marker genes were displayed with Cytoscape software.

Drugs that may target the diagnostic markers were predicted through the DGIdb database. The interactions between drugs and markers were visualized with Cytoscape software (Figure 6). Altogether 25 drugs that target markers were predicted from DGIdb database, which including 11 for CXCR4, 6 for CD19, 3 for IL2RG, and 5 for FOS. However, no drugs were predicted for other three genes (FABP4, IGHD, and PPBP).

## Discussion

Even though periodontitis is caused by bacteria that colonize the surface of teeth and the gingival sulcus, the host response is believed to play a key role in the destruction of periodontal connective tissue and alveolar bone. Bacteria trigger an immune response in the host, leading to local inflammatory infiltration and resorptive activity by osteoclasts, ultimately resulting in severe damage to the periodontal tissue.

Similar to a previous study, we identified three types of immune cells (naïve B cells, neutrophils, and plasma cells elevated in periodontitis samples (28). Plasma cells make up approximately 50% of total immune infiltrates (29, 30). The data suggested the accuracy of the CIBERSORT algorithm in predicting the percentage of infiltrating immune cells in the gingival microenvironment.

Neutrophils (PMNs) exert bidirectional effects on inflammation in chronic periodontitis. On the one hand, the bactericidal function of PMNs can control infections by lowering the level of pathogenic microorganisms and thus inhibiting inflammatory responses (31). On the other hand, PMNs are activated and recruited to the site of infection when bacteria and their products invade the host, its metabolic products lead to imbalance of periodontal homeosta, causing destruction of periodontal supportive tissues, while the active oxygen species produced by it cause bacterial- and host-mediated damage to periodontal tissues (32). PMNs can also promote bone resorption by upregulating the production of pro-inflammatory factors, activating the expression of RANKL, or even directly acting on osteoclasts to promote bone loss to promote alveolar bone resorption (7, 33).

Lymphocytes also play a dual role in chronic periodontitis, both accelerating the destruction of periodontal tissues (34, 35) and inhibiting periodontal inflammation. When considering the role of lymphocytes, it is necessary to consider specific conditions and specific lymphocyte subtypes.

The host's inflammatory immune system plays a dual role in chronic periodontitis, with numerous genes or cytokines playing different roles in the process. Controlling periodontal infections and destroying periodontal tissues share similar cellular and molecular signaling pathways. Cytokines are considered an intermediate mechanism between bacterial stimulation and tissue destruction (36). The balance between stimulatory and inhibitory cytokines, as well as the regulation of their receptors and signal cascades, determines the level of tissue loss in the periodontal tissue.

In this study, seven genes (CD19, CXCR4, FABP4, FOS, IGHD, IL2RG, and PPBP) were significantly up-regulated in periodontitis samples. Altogether 25 drugs that target markers were predicted from DGIdb database, which including 11 for CXCR4, 6 for CD19, 3 for IL2RG, and 5 for FOS. CD19 is a B cell-specific transmembrane protein that is expressed during the pre-B cell stage to the plasma cell differentiation stage in humans and mice, and functions as a co-receptor of the B cell receptor (37). CD19 is essential at multiple stages of B cell development, and its expression level and activity directly affect the growth, differentiation, and function of B cells. CXCR4, one of the marker genes, is also an important chemokine receptor. Chemokines are a kind of cytokines that mediate the recruitment and activation of leukocytes and participate in the pathogenesis of various immune system-related diseases, including periodontitis (38, 39). The interleukin 2 receptor subunit gamma chain (IL2Rg, also known as CD132) is a common receptor subunit for several important immune factors, including IL-2, IL-4, and IL-7, among others. The FOS gene, a component of the AP-1 transcription factor complex, is implicated in various cellular processes, including cell proliferation, differentiation, and survival (40). Some studies have found that an appropriately high expression of AP1 can enhance the body's immune and antibacterial ability and improve its ability to respond to multiple stimuli (41). The AP-1 family of transcription factors can activate Toll-like receptor agonists and positively regulate interleukin-4 (IL4) to activate macrophages, thereby enhancing antibacterial activity (42). Fos/AP-1 also plays a central role ininflammatory bone loss by regulating genes like NFATc1 as well as the interferon system (43).

Whilst antimicrobial strategies might potentially contribute to the management of periodontitis, a growing awareness prevails that irreversible tissue damage is mainly ascribed to host responses. This has impelled numerous researchers to direct their attention towards strategies targeting host signaling pathways. Despite the fact that the precise mechanisms underlying the onset and persistence of periodontitis remain incompletely comprehended, there is already adequate knowledge at the experimental level to furnish valuable information for targeted therapeutic interferences. The successful interventional measures in animal models have targeted various interrelated inflammatory pathways, such as the recruitment of inflammatory cells, complement activation, pro-inflammatory cytokines, and RANKL-dependent osteoclastogenesis (4). Although we identified dozens of drugs that target the immune-related marker genes in our study, there was no study to support the efficacy of these drugs in periodontitis. Further studies are needed to investigate the efficacy and mechanism of these drugs in the treatment of periodontitis. In our study, we identified 7 IRG as diagnostic markers and constructed a risk model based on these 7 genes. These marker genes are involved in the regulation of the immune microenvironment in periodontitis samples. Our future studies will focus on the underlying mechanism of these marker genes and targeting drugs for periodontitis.

## Data Availability

The datasets presented in this study can be found in online repositories. The names of the repository/repositories and accession number(s) can be found below: https://www.ncbi.nlm.nih.gov/, Gene Expression Omnibus.

## Supplementary material

Supplementary Figure S1 The expression levels of 7 DE-IRGs were displayed in heatmap plot.
