# Supplementary figures and images for: Genetic analysis of potential markers and therapeutic targets for immunity in periodontitis
Source: Front Dent Med. 2024 Nov 22;5:1480346. doi: 10.3389/fdmed.2024.1480346 (PMC11797874; doi:10.3389/fdmed.2024.1480346)

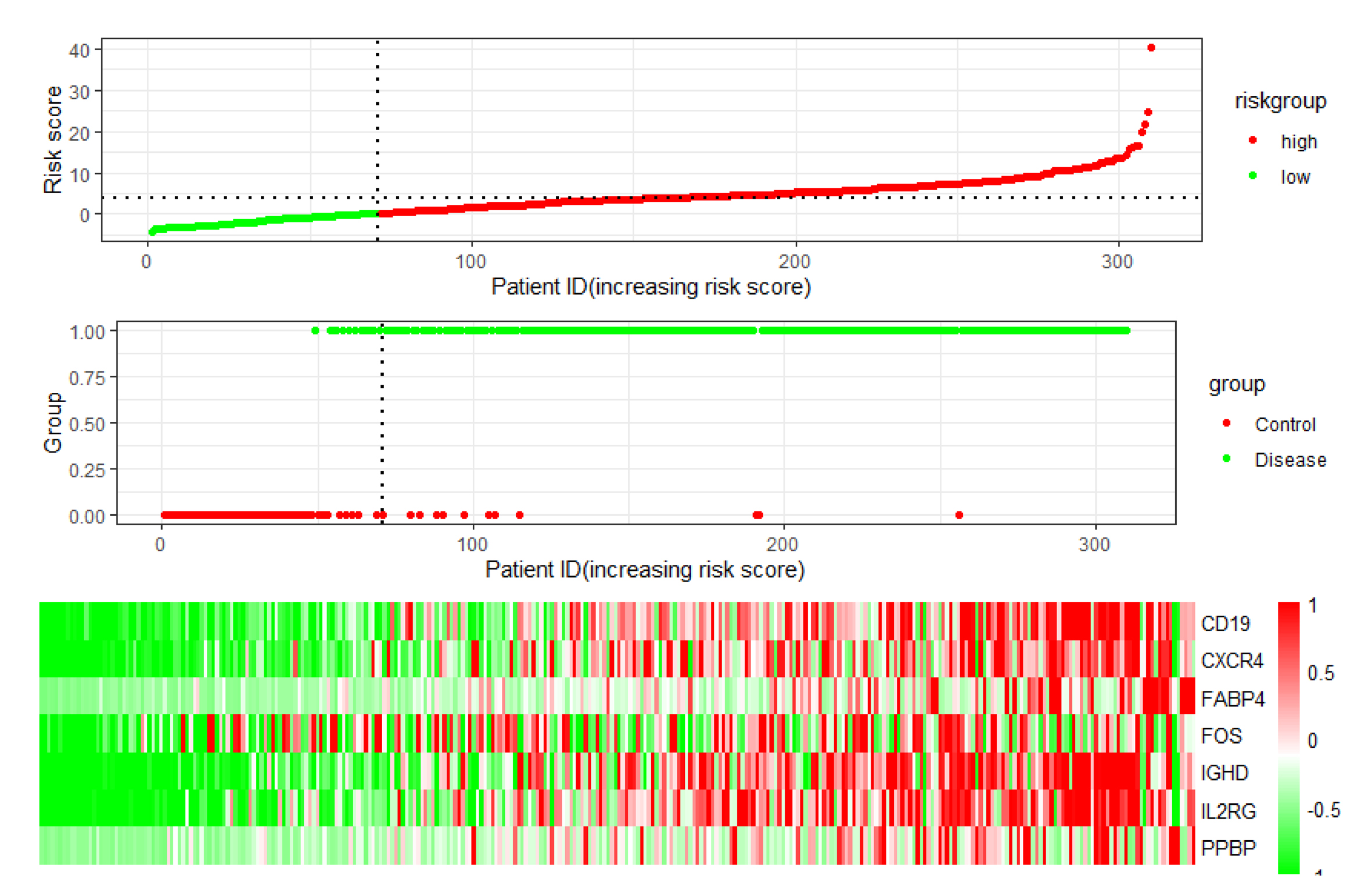

Supplement: Supplementary Figure S1 — The expression levels of 7 DE-IRGs were displayed in heatmap plot. [file Image1.jpeg]
